# Solvent-Dependent Fluorescence Behavior and Water Detection Sensor Application of Visible Light-Emitting Fluorenone Derivative

**DOI:** 10.1007/s10895-023-03531-6

**Published:** 2023-12-12

**Authors:** Jineun Lee, Heesang Kim, Toshikazu Sakaguchi, Giseop Kwak

**Affiliations:** 1 Department of Polymer Science & Engineering, Polymeric Nanomaterials Laboratory, Kyungpook National University, 1370 Sankyuk-dong, Buk-ku, Daegu, 702–701 South Korea; 2 Department of Materials Science and Engineering, Graduate School of Engineering, University of Fukui, Bunkyo 3-9-1, Fukui, 910-8507 Japan

**Keywords:** Fluorenone, Intramolecular charge transfer, Vibronic coupling, Solvatochromism, Fluorescence quenching

## Abstract

The Sonogashira coupling reaction was used to synthesize a fluorenone derivative, with an extended conjugated structure to which fluorene is connected via acetylene linkage. This compound exhibited diverse fluorescence (FL) colors in the visible region depending on the polarity of the matrix solvents used. The solvatochromic FL presented as sky blue, green, and yellow in hexane, THF, and DMF, respectively. Fluorene moiety and fluorenone moiety acted as an electron donor (D) and as an electron acceptor (A), respectively, leading to an excited state intramolecular charge transfer based on the D–π–A electronic structure. In particular, this derivative showed a remarkable FL quenching in alcohol and chloroform, probably due to vibronic coupling through hydrogen bonding with these solvents. This idea was supported by the fact that the two solvents are characterized by very high hydrogen bond donor acidities compared to other solvents used in this study. This derivative also responded to the presence of very small amounts of water at several mg/mL levels in organic solvents, resulting in remarkable FL quenching.

**Supplementary Information:**

The online version contains supplementary material available at 10.1007/s10895-023-03531-6.

## Introduction

Conjugated π systems are not only used as core materials for optoelectronic devices, such as organic light-emitting diodes and organic photovoltaic solar cells, but are also being applied as fluorescent sensors that sensitively respond to external stimuli, such as temperature and chemicals [1–4]. It is very important to thoroughly understand their photophysical properties in developing them into advanced optoelectronic materials [5]. Fluorenone is particularly interesting to hold a potential application as a sensor due to its very unique fluorescence (FL) behavior [6–9]. Fluorenone exhibits significant FL quenching in alcohol, which can be explained as a phenomenon due to vibronic coupling caused by alcohol molecules [10, 11]. FL quenching appears very dramatically; therefore, it can be said to be an inherent photophysical property of fluorenone compared to other fluorophores. However, since fluorenone does not have a sufficiently extended conjugation length, the electron transition occurs only in ultraviolet region; thus, FL quenching cannot be recognized with the naked eye, which may be a drawback for sensor application.

This study attempted to address this issue and further explore new sensor applications. Organic compounds with an extended conjugated structure composed of aromatic and unsaturated hydrocarbon groups can exhibit FL emission in the visible region mainly due to π–π* electronic transition [12]. This study used the Sonogashira coupling reaction to synthesize a fluorenone derivative that can exhibit FL in the visible region by connecting fluorenone and fluorene groups to each other via acetylene linkage. FL behavior was investigated in various matrix solvents for this extended π-conjugation system. It was identified that due to intramolecular charge transfer and vibronic coupling, the fluorenone derivative exhibited not only a remarkable solvatochromic FL but also FL quenching, depending on physicochemical properties of the solvents, such as polarity and hydrogen bond donor (**HBD**) acidity. Based on such a solvent-dependent FL behavior, the fluorenone derivative responded to a very small amount of water in organic solvents, resulting in considerable FL quenching.

## Material and Method

### Materials

Fluorene, iodomethane, 2-methyl-3-butyn-2-ol, iodine, triphenylphosphine, bis(triphenylphosphine)palladium(II) dichloride, copper(I) iodide, and the organic solvents were obtained from Fujifilm Wako Pure Chemical Co., Ltd and were used as received without further purification. All the solvents were stored in a dry state using anhydrous sodium sulfate until immediately before use. These compounds were used to synthesize 2-ethyl-9,9-dimethylfluorene according to the literature [13]. 2-Bromo-9-fluorenone was obtained from Tokyo Chemical Industry Co., Ltd.

### Synthesis of 1-(Fluorenone-2-yl)-2-(9,9-dimethylfluorene-2-yl)Acetylene (FDMFA)

A 500-mL round-bottom three-neck flask was equipped with a reflux condenser, a three-way stopcock, and a magnetic stirring bar and was flushed with dry nitrogen. 2-Bromo-9-fluorenone (11.7 g, 45 mmol), bis(triphenylphosphine)palladium dichloride (0.112 g, 0.16 mmol), cupper iodide (0.183 g, 0.96 mmol), triphenylphosphine (0.168 g, 0.64 mmol), and piperidine (250 mL) were placed in the flask. Subsequently, 2-ethynyl-9,9-dimethylfluorene (9.82 g, 45 mmol) in piperidine (50 mL) solution was added, and the reaction mixture was stirred at 90 °C for 24 h. After the piperidine in the reaction mixture was evaporated, ether (~ 500 mL) was added, and the insoluble salt was then filtered off. The filtrate was washed with 1 M hydrochloric acid and then with water. The solution was dried over anhydrous sodium sulfate and then concentrated at reduced pressure. Silica gel column chromatography (eluent: hexane/chloroform = 2:1) was performed to purify the crude product, obtaining the desired yellow solid product (9.32 g, 52% yield).

^1^H Nuclear magnetic resonance (NMR) (500 MHz, CDCl_3_, ppm): 7.81 (s, 1H, ArCH), 7.75–7.62 (m, 5 H, ArCH), 7.54–7.41 (m, 5 H, ArCH), 7.36–7.25 (m, 3 H, ArCH), 1.51 (s, 6 H, CH_3_). ^13^C NMR (125 MHz, CDCl_3_, ppm): 193.0, 153.9, 153.6, 143.9, 143.5, 139.7, 138.4, 137.5, 134.8, 134.3, 134.2, 130.8, 129.3, 127.8, 127.2, 127.1, 125.9, 124.4, 124.3, 122.6, 121.1, 120.5, 120.31, 120.27, 120.0, 92.0, 88.7, 46.9, 27.0. HR–MS (DART): for C_30_H_21_O (M + H)^+^ (calc.: 397.1592; found: 397.1582).

### Measurements

NMR spectra were recorded on ECX-500 (JEOL, Japan) spectrometer at room temperature with CDCl_3_ as the solvent. Mass spectra were recorded on an AccuTOF-DART (JEOL, Japan) mass spectrometer, with poly(ethylene glycol) (MW: 400) used for the calibration. Ultraviolet–visible (UV–Vis) absorption spectra were recorded on a JASCO V-650 spectrophotometer at room temperature, and the FL spectroscopy was performed on a JASCO FP-6500 spectrofluorometer at an excitation wavelength of 350 nm. The FL quantum efficiencies (**FLQE**s) of **FDMFA** in various solvents were determined relative to a quinine sulfate solution in a 0.5-M H_2_SO_4_ at room temperature, assuming a quantum yield of 0.546 when excited at 365 nm.

### Test for Detection of Alcohols and Water in THF

FL emission spectra were measured upon adding alcohol and water as an analyte to the THF solution of **FDMFA**.

The Stern–Volmer constant (*K*_sv_) was determined by plotting the FL intensity change as a function of the molar concentration of the analyte according to the following equation [14].

1 $$\frac{{F}_{0}}{F}=1+{K}_{SV}\left[Q\right]$$

where *F*_0_ and *F* are the initial FL intensity without the analyte and the FL intensity with the analyte, respectively, and [*Q*] is the analyte molar concentration.

Similarly, limit of detection (**LOD**) and limit of quantitation (**LOQ**) were determined by plotting the FL intensity change as a function of the weight concentration of the analyte according to the following equations [15]:

2 $$LOD=\frac{3.3\sigma }{S}$$

3 $$LOQ=\frac{10\sigma }{S}$$

where *σ* and *S* are the standard deviation of y-intercept and slope of the calibration curve, respectively.

**Fig. 1:**
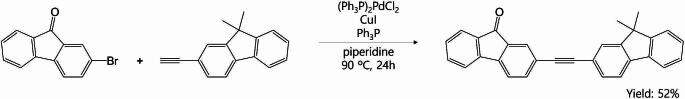
Synthetic route of **FDMFA**.

**Fig. 2:**
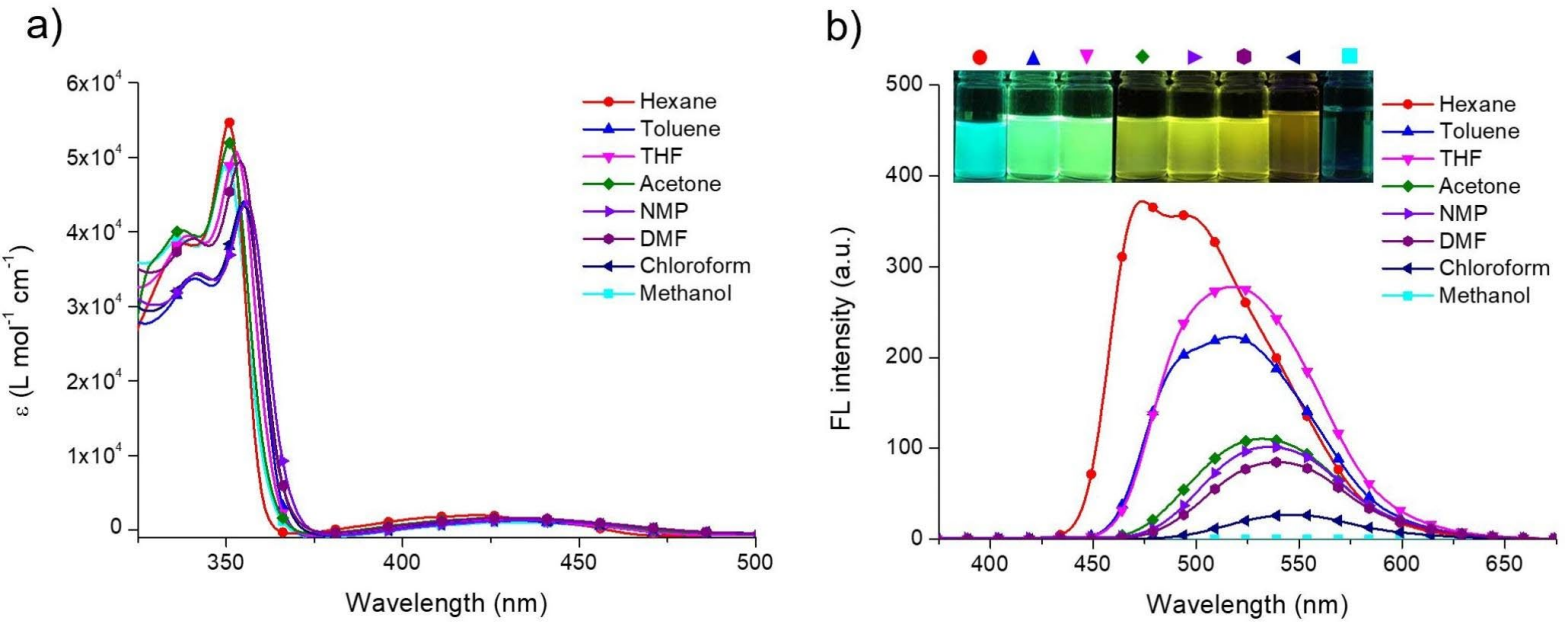
(a) UV–Vis absorption and (b) FL emission spectra of 1.0 × 10^− 5^ M **FDMFA** solution in various solvents when excited at 350 nm

## Results and Discussion

To impart an extended conjugated structure to fluorenone, a derivative, in which the fluorene group is connected via acetylene linkage, was synthesized through the Sonogashira coupling reaction (**FDMFA** in Fig. 1). The chemical structure was identified by ^1^H and ^13^C NMR spectra (Fig. S1). UV–Vis absorption and FL emission spectra of this compound were measured in various solvents (Fig. 2). **FDMFA** showed a maximum absorption at around 350 nm and a relatively weak and broad absorption band around 425 nm (Fig. 2a). Table 1 summarizes the spectroscopic results. These two characteristic bands are due to π–π* and n–π* electronic transitions, respectively. In particular, in nonpolar hydrocarbon (hexane), aprotic polar (acetone), and protic polar (methanol) solvents, the absorption band appeared at a relatively shorter wavelength and shifted to a longer wavelength in aprotic polar solvents (DMF, NMP) and nonpolar aromatic solvent (toluene). This indicates that the interaction with the matrix molecules diversely affects the electronic energy level of **FDMFA** in the ground state.

**Fig. 3:**
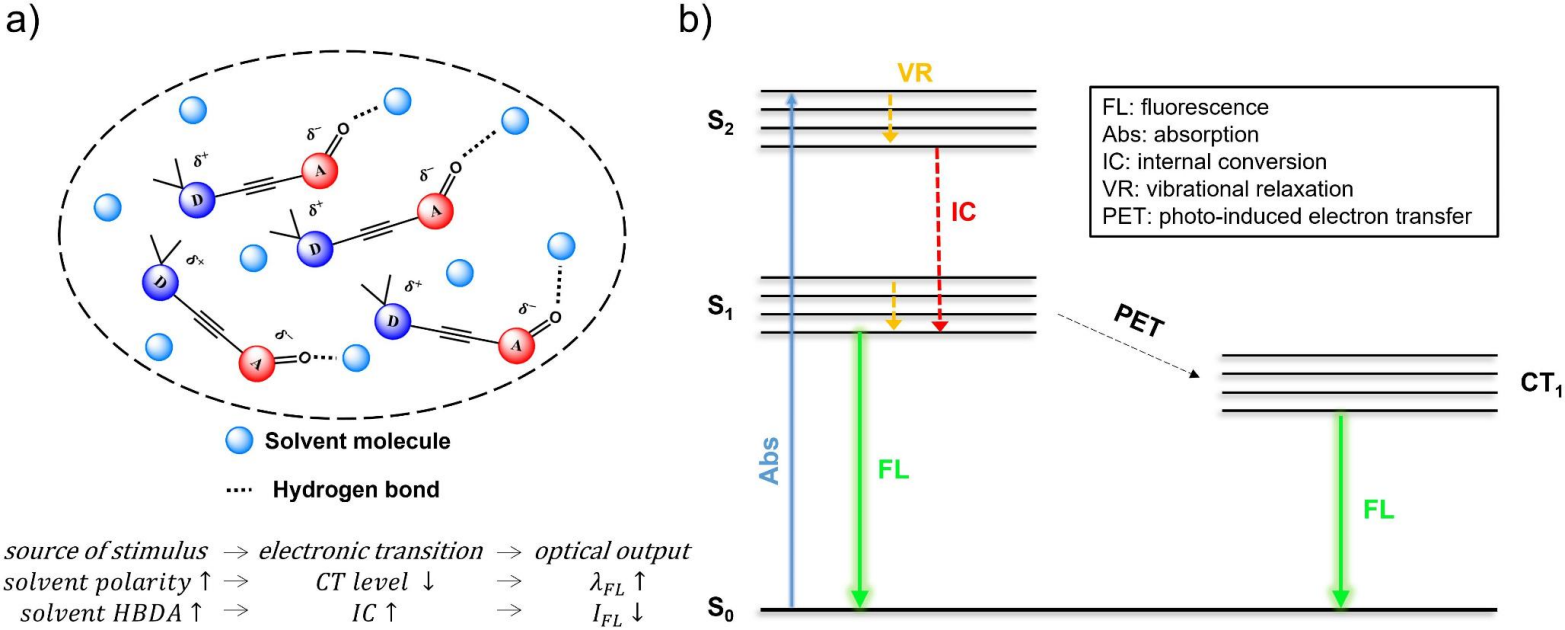
(a) Schematic illustration (↑ and ↓ indicate “increase” and “decrease,” respectively) and (b) Jablonski energy diagram on electronic transition change by interaction between FDMFA and solvent

On the other hand, **FDMFA** showed an FL behavior that highly depends on the polarity (dipole moment, dielectric constant) of the matrix solvent (Fig. 2b, Table 1). This compound showed maximum FL intensity at 474 nm in hexane, a nonpolar aliphatic hydrocarbon compound. However, as the polarity of the solvent increased, the compound underwent a large bathochromic shift to show maximum FL at 518 and 539 nm in THF and in DMF, respectively. Consistent with this spectral result, **FDMFA** presented as sky blue, green, and yellow in hexane, THF, and DMF, respectively (inset of Fig. 2b). Simultaneously with the red shift, FL intensity decreased significantly, corresponding to a typical solvatochromic FL, which indicates the occurrence of an intramolecular charge transfer in the excited state. This should be due to a donor–π–acceptor (D–π–A) electronic structure for the following reasons. Since **FDMFA** is all composed of sp^2^ and sp hybridized carbons, coplanarity is very high for the molecular geometry; therefore, the π electrons are estimated to be highly delocalized throughout the overlapped p orbitals in the molecule [12]. Accordingly, the fluorenone moiety can act as an electron acceptor (A) because the carbonyl group serves as a strong electron-withdrawing group, while the π electron-rich fluorene moiety acts as the electron donor (D) (Fig. 3a) [16]. Notably, in methanol and chloroform, the FL of **FDMFA** seemed to be greatly affected by another factor in addition to charge transfer. **FDMFA** showed exceptionally large bathochromic shift and FL quenching in these two solvents even though these solvents are relatively less polar than DMF. This was quite different from the typical FL behavior due to intramolecular charge transfer. FL quenching in methanol occurred critically, so the **FLQE** was close to zero, and it was difficult to identify the maximum FL wavelength from the very weak band. This should be due to the vibronic coupling caused by methanol molecules even in the present fluorenone derivative with an extended conjugated structure [6–9]. Similarly, **FDMFA** showed a very large bathochromic shift even in chloroform, displaying a maximum FL at 547 nm, which was shifted to a longer wavelength by 8 nm compared to that in DMF with higher polarity. FL was significantly attenuated even in chloroform. Moreover, the **FLQE** was determined to be 1.6%, corresponding to only 1/8–1/10 of the quantum efficiencies in hexane, toluene, and THF and about 1/3–1/4 of quantum efficiencies in NMP and DMF. Accordingly, chloroform can also be said to cause vibronic coupling to **FDMFA**, resulting in significant FL quenching. Even when the excitation wavelength was changed in each solvent, only the FL intensity of FDMFA changed, but the maximum FL wavelength and band shape did not change (Fig. S2). This indicates a single excited species. Moreover, similar solvent dependence as above was shown at other excitation wavelengths (Fig. S3). The succeeding description thoroughly discusses the intramolecular charge transfer and vibronic coupling phenomena on the FL solvatochromism and quenching of **FDMFA** with the Jablonski energy diagram.

**Table 1:** Properties of solvents used in this study and the spectral results of FDMFA measured in these solvents

Solvents	Properties				Spectral results		
	µ^a^	ε^b^	α_KT_^c^		λ_abs,max_^d^ (nm)	λ_FL,max_^d^ (nm)	**FLQE** ^e^ (%)
n-Hexane	0.08	1.88	0.00		351, 422	474, 494	15.3
Toluene	0.31	2.38	0.00		355, 428	495, 518	16.6
THF	1.75	7.58	0.00		353, 428	518	13.9
Acetone	2.69	20.7	0.08		351, 428	532	4.50
NMP	4.09	32.2	0.00		356, 434	536	6.94
DMF	3.86	36.7	0.00		354, 433	539	4.40
Chloroform	1.15	4.81	0.44		355, 433	547	1.61
Methanol	2.87	32.7	0.93		350, 430	N/D	~ 0

^a^Dipole moment from solvent handbook. ^b^Dielectric constant from solvent handbook. ^c^**HBDA** values from ref. 18. ^d^Determined in **FDMFA** dilute solution (1.0 × 10^− 5^ M) at room temperature. ^e^Determined as described in experimental section.

The unique FL behavior of **FDMFA** based on “intramolecular charge transfer” and “vibronic coupling” phenomena is highly related to non-covalent interactions with matrix solvent molecules. That is, the higher the polarity of the matrix solvent, the greater the polarization of **FDMFA**, resulting in a lower charge transfer level (CT_1_) in the excited state, as shown in Fig. 3. Consequently, a significant FL redshift appears along with the matrix solvent polarity. In particular, methanol causes intramolecular charge transfer of **FDMFA** and simultaneously generates internal conversion in the electronic transition through vibronic coupling. This leads to significant non-radiative decay in addition to FL redshift. The carbonyl group of fluorenone moiety has a large dipole moment and a partially negative charge on the oxygen atom [17]. Therefore, **FDMFA** can intrinsically act as a hydrogen bond acceptor in hydrogen bonding with matrix molecules (Fig. 3a) [18, 19]. During this time, the matrix molecule is expected to act as an **HBD**. For this reason, the hydrogen bond donor acidity (**HBDA**) of solvent molecules will affect the vibronic coupling caused by the intermolecular interaction. That is, the higher this value, the more pronounced the FL quenching. As mentioned previously, the **FLQE** reached almost zero in a matrix with a very high **HBDA**, such as alcohol (0.93 for methanol in Table 1). On the other hand, in hydrocarbon (hexane, toluene), ether (THF), and amide (NMP, DMF) solvents, which all have zero **HBDA** value, solvatochromism appears more dominant than FL quenching. Therefore, it can be said that in these nonalcoholic solvents, the intramolecular charge transfer has a more dominant effect on the FL behavior of **FDMFA**. It should also be noted that although chloroform is a nonalcoholic solvent, the FL quenching was exceptionally remarkable. This can be attributed to chloroform’s much higher **HBDA** value (0.44 for CHCl_3_) relative to other nonalcoholic solvents. This high **HBDA** comes from an induction effect, which is due to the high electronegativity of chlorine atom.

**Fig. 4:**
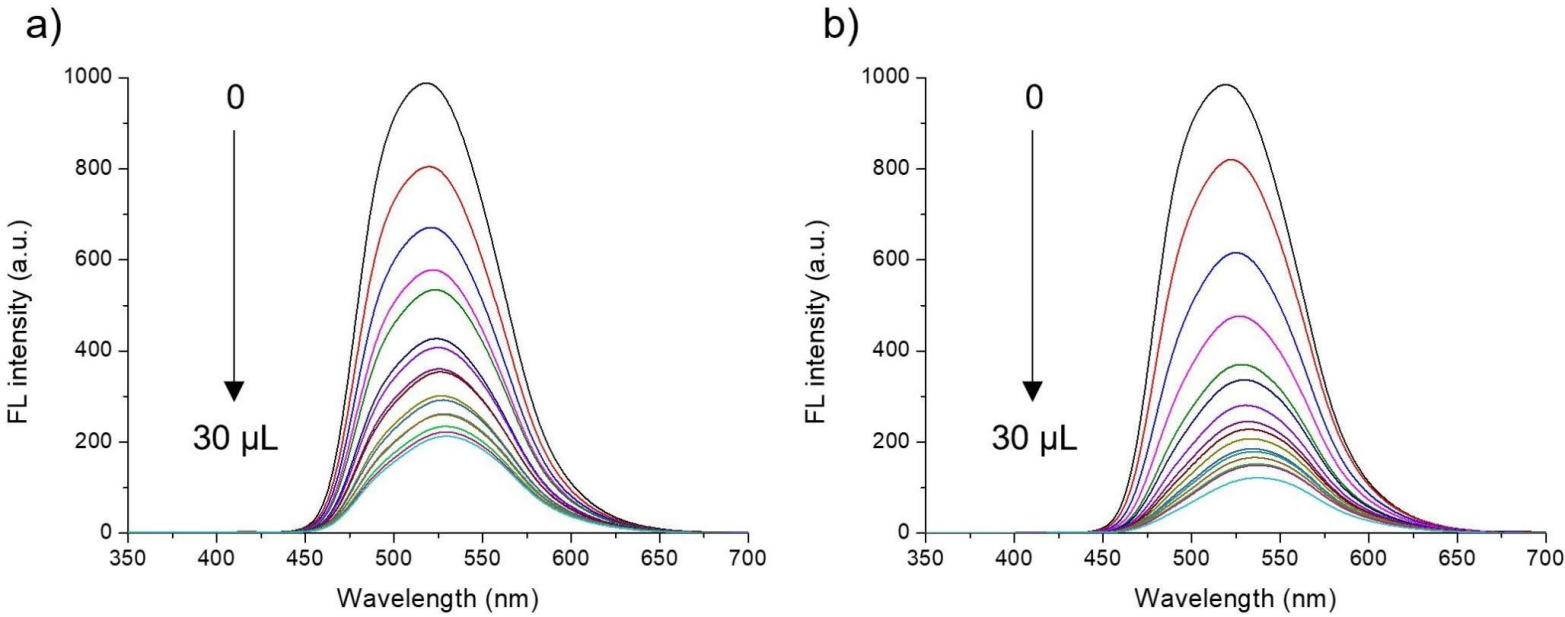
FL emission spectra upon addition of (a) methanol and (b) water to a solution of 1.0 × 10^− 5^ M **FDMFA** in THF (excited at 350 nm, adding 2 µL each up to 30 µL)

**Table 2:** Test results for the detection of alcohols and water in THF using FL quenching of FDMFA

Analytes	α_KT_^a^	K_sv_^b^ (mol^− 1^∙L)	LOD^c^ (mg/mL)	LOQ^c^ (mg/mL)
Methanol	0.93	10.07	0.40	1.23
Ethanol	0.83	11.00	0.32	0.97
1-Propanol	0.78	11.02	0.54	1.64
n-Butanol	0.69	9.92	0.60	1.81
Water	1.17	7.74	0.43	1.30

^a^**HBDA** values from ref. 18. ^b^Stern–Volmer constant determined as described in experimental section. ^c^Determined as described in experimental section.

**FDMFA** has an extended D–π–A conjugated structure composed of fluorenone acceptor, acetylene linkage, and fluorene donor. **FDMFA** exhibits FL in the visible region and is readily dissolved in common organic solvents due to the two methyl groups attached to the fluorene moiety. Therefore, the FL variation caused by the non-covalent interaction with the matrix molecules can be easily recognized with the naked eye. In this respect, **FDMFA** holds potential as a probe material. To demonstrate this, the FL spectral change according to the methanol content in THF was investigated (Fig. 4a). As the methanol content increased, the FL intensity decreased as expected. Moreover, it was possible to detect methanol in THF at several mg/mL levels. Other alcohols were also examined for the same test (Fig. S4). Similar FL quenching was observed in all the alcohols, although there was no tendency along with the alkyl length of the alcohol molecule. The Stern–Volmer constants were all quite high (> 9.9 mol^− 1^∙L) (Table 2, Fig. S5). This indicates that the vibronic coupling due to hydrogen bonding between **FDMFA** and alcohol occurs regardless of the size of the alcohol molecule. The **LOD** and **LOQ** were quite low (0.40 and 1.23 for methanol, 0.32 and 0.97 for ethanol, 0.54 and 1.64 for n-propanol, and 0.60 and 1.81 for n-butanol, respectively) (Table 2, Fig. S6).

Organic solvents often absorb moisture from the air during manufacturing and used to be contaminated. Accordingly, water in organic solvents acts as an impurity in most chemical reactions, causing a decrease in yield and purity of the product. Fluorescent sensor materials that can detect moisture in organic solvents with a high sensitivity have recently been developed [20–24]. It is expected that water, like alcohols, can act as **HBD** and cause FL quenching. Therefore, **FDMFA** was also evaluated for its ability to detect water in organic solvents. As in the case of alcohol, significant FL quenching was observed as the water content in THF increased (Fig. 4b). Since water is a non-solvent for **FDMFA**, it might cause aggregation when present in a high content. In spectroscopy, an isosbestic point is a specific wavelength at which the total absorbance of a sample does not change during a chemical reaction or a physical change of the sample. In our case, if aggregation occurs during the process of adding water, the total absorbance of FDMFA and its complex (FDMFA-water) will change and the isosbestic point will not appear. However, isosbestic point was observed at 422 nm in the absorption spectra even when the water content increased. This indicates that aggregation did not occur at several mg/mL levels examined in this study (Fig. S7). Therefore, FL quenching is not due to the formation of excimers by aggregation but is due to vibronic coupling by water molecules. This idea is supported by the fact that water has an extremely high **HBDA** of 1.17 [25–27]. **FDMFA** still has a quite high Stern–Volmer constant of 7.74 for the detection of water in THF, despite being slightly lower than those for the detection of alcohols (Table 2, Fig. S5). The **LOD** and **LOQ** were also quite low, which are determined to be 0.43 and 1.30, respectively (Table 2, Fig. S6). This indicates the possibility of use as an FL sensor to detect water in various organic solvents.

## Conclusion

The Sonogashira coupling reaction was used to synthesize **FDMFA** that is composed of fluorenone and fluorene, which serve as electron acceptor and electron donor, respectively, and are connected via acetylene linkage. This compound exhibited a variety of visible light FL colors in matrix solvents. The solvatochromic FL was thought to result from the intramolecular charge transfer in an excited state based on the D–π–A electronic structure. In particular, **FDMFA** showed a remarkable FL quenching in alcohol and chloroform, which was thought to be due to the vibronic coupling caused by the solvent molecules through hydrogen bonding. In fact, these two solvents have quite high **HBDA** values compared to other solvents used in this study. This meant that the two solvents greatly caused internal conversion via vibronic coupling, leading to non-radiative decay. **FDMFA** also responded to a small amount of water at several mg/mL levels in organic solvents, resulting in FL quenching. Consequently, the present fluorenone derivative can be used as a fluorescent probe sensor capable of detecting water in various organic solvents.

## Electronic Supplementary Material

Below is the link to the electronic supplementary material.

Supplementary Material 1

## Data Availability

The datasets generated during the current study are available from the corresponding author on reasonable request.
