# Supplementary material for: Solvent-Dependent Fluorescence Behavior and Water Detection Sensor Application of Visible Light-Emitting Fluorenone Derivative
Source: J Fluoresc. 2023 Dec 12;35(1):437–43. doi: 10.1007/s10895-023-03531-6 (PMC11807051; doi:10.1007/s10895-023-03531-6)
Supplement: Supplementary file 1 — Supplementary Material 1 [file 10895_2023_3531_MOESM1_ESM.docx]

*Supporting Information*

Solvent-Dependent Fluorescence Behavior and Water Detection Sensor Application of Visible Light-Emitting Fluorenone Derivative

Jineun Lee, *^†^* Heesang Kim, *^†^* Toshikazu Sakaguchi, *^‡,^** Giseop Kwak*^†,^**

*^†^*Department of Polymer Science & Engineering, Polymeric Nanomaterials Laboratory, Kyungpook National University, 1370 Sankyuk-dong, Buk-ku, Daegu 702–701, South Korea

*^‡^*Department of Materials Science and Engineering, Graduate School of Engineering,
University of Fukui, Bunkyo 3-9-1, Fukui 910-8507, Japan

KEYWORDS: fluorenone, intramolecular charge transfer, vibronic coupling, solvatochromism, fluorescence quenching


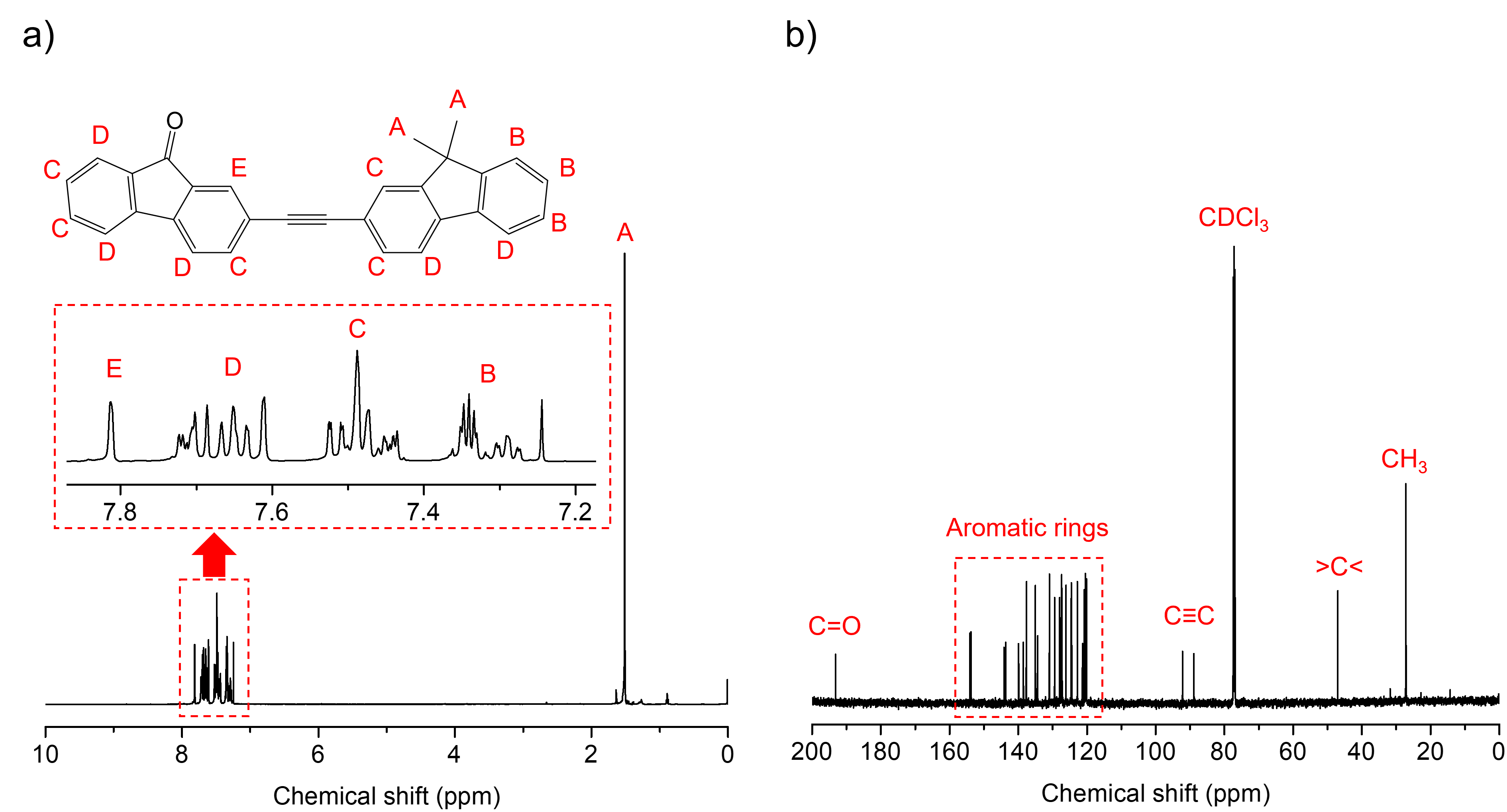


**Fig. S1.** a) ^1^H and b) ^13^C NMR spectra of **FDMFA**.


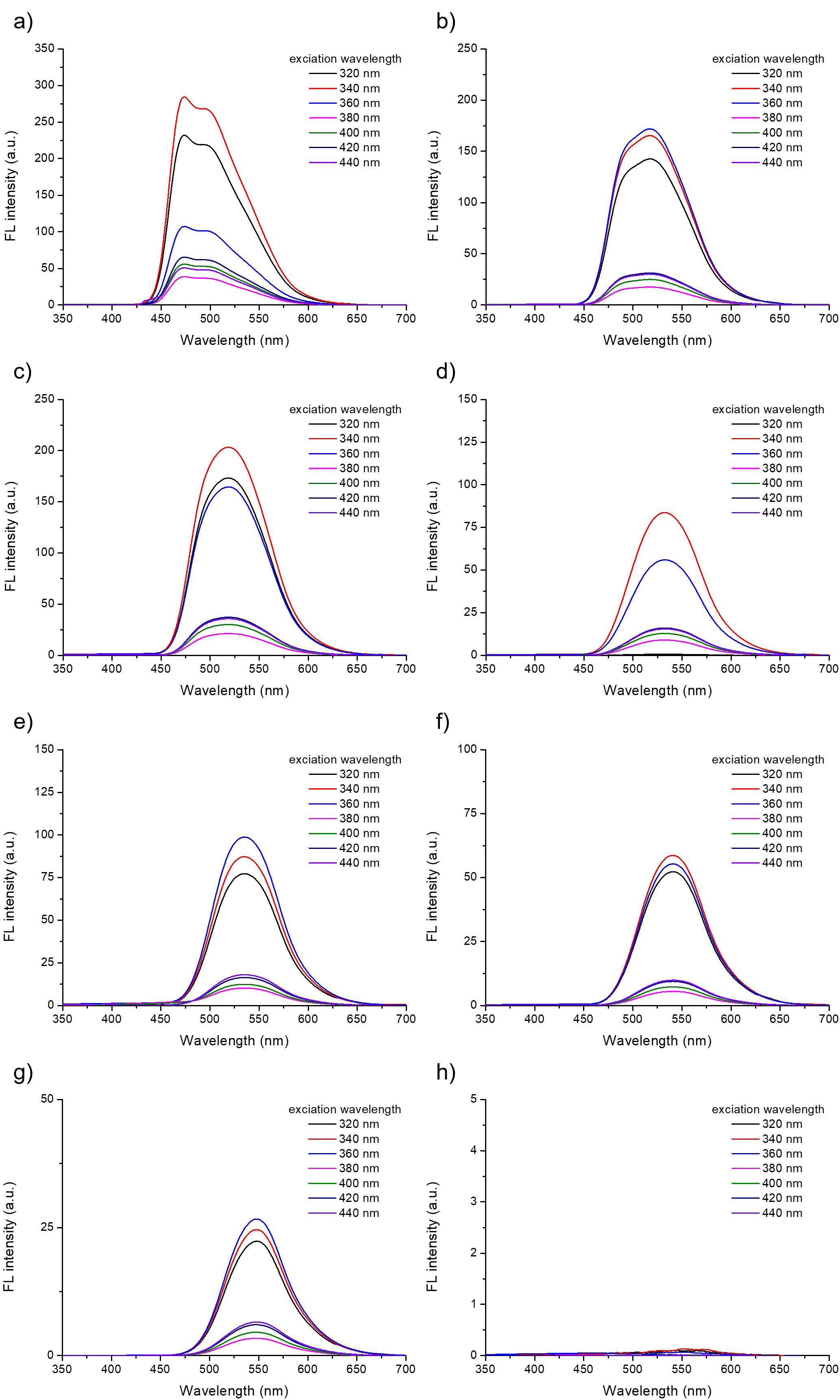


**Fig. S2.** FL emission spectra of 1.0 × 10^-5^ M **FDMFA** solution in a) hexane, b) toluene, c) THF, d) acetone, e) NMP, f) DMF, g) chloroform, and h) methanol at different excitation wavelengths.

**Fig. S3.** FL emission spectra of 1.0 × 10^-5^ M **FDMFA** solution in various solvents when excited at 425 nm.


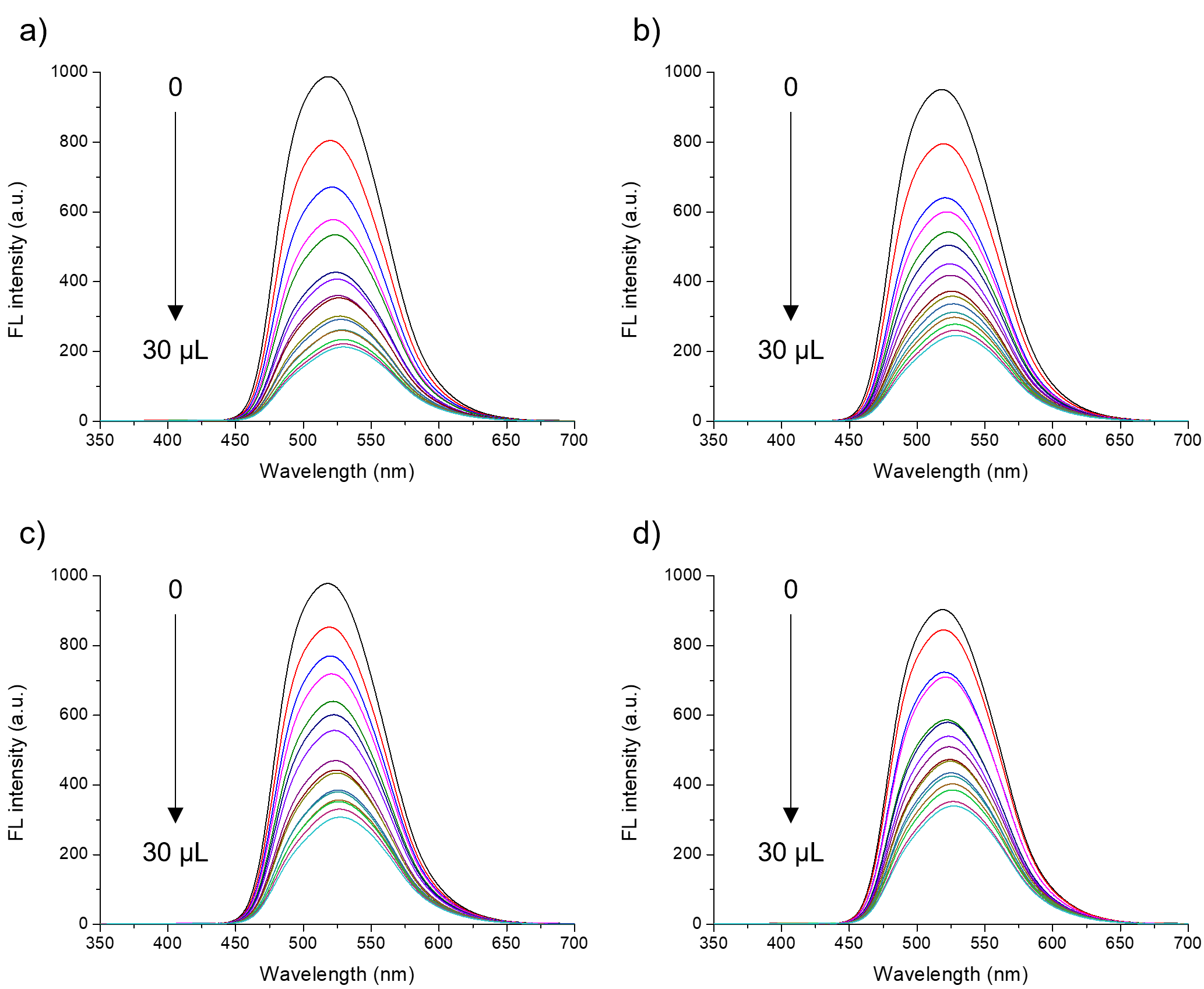


**Fig. S4.** FL emission spectra upon addition of a) methanol, b) ethanol, c) 1-propanol, and d) n-butanol to a solution of 1.0 x 10^-5^ M **FDMFA** in THF (adding 2 μL each up to 30 μL).


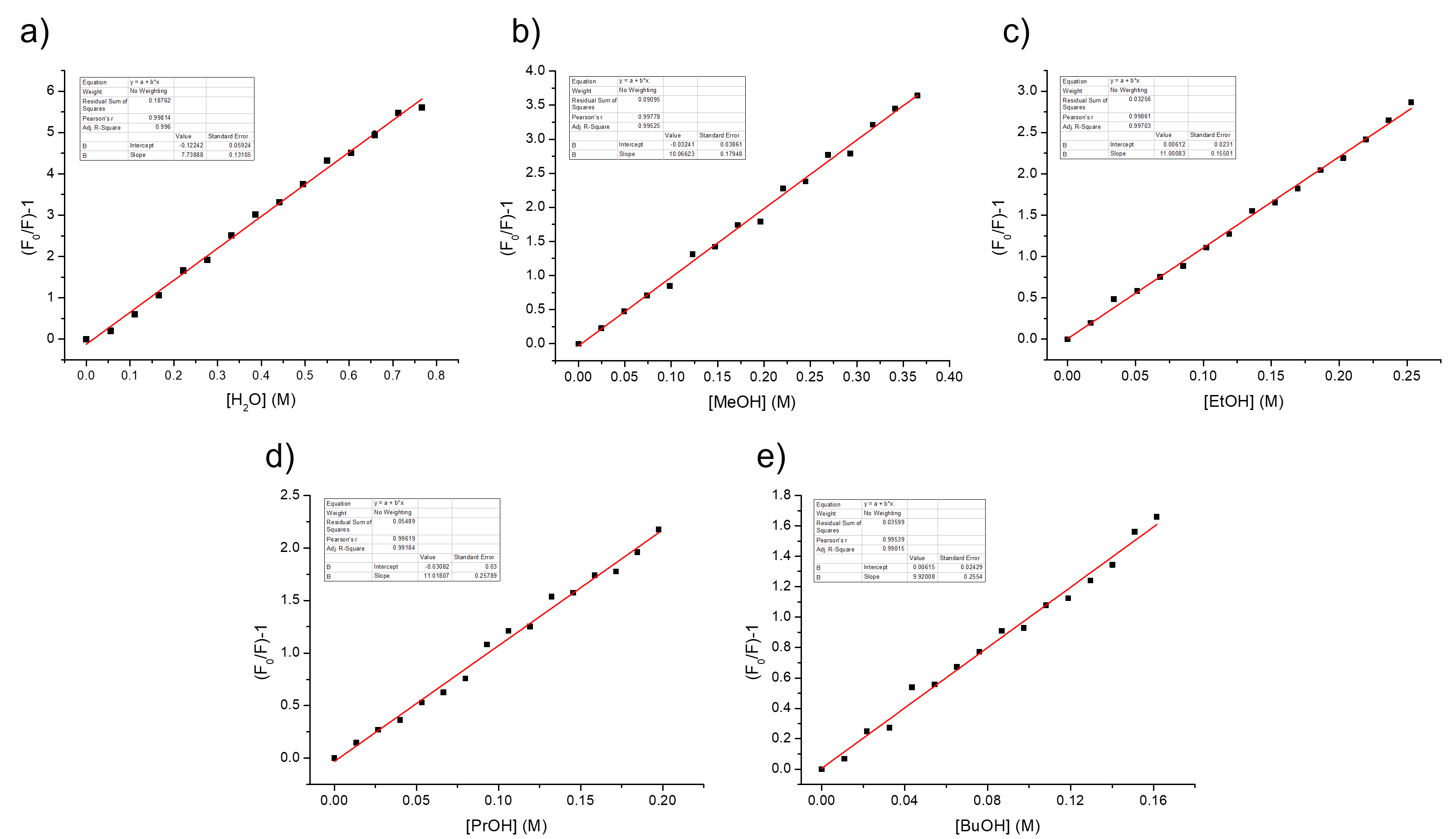


**Fig. S5.** Stern-Volmer plots for the FL quenching of **FDMFA** by a) water, b) methanol, c) ethanol, d) 1-propanol, and e) n-butanol.

**Fig. S6.** Plot for the FL quenching of FDMFA by water, methanol, ethanol, 1-propanol, and n-butanol.


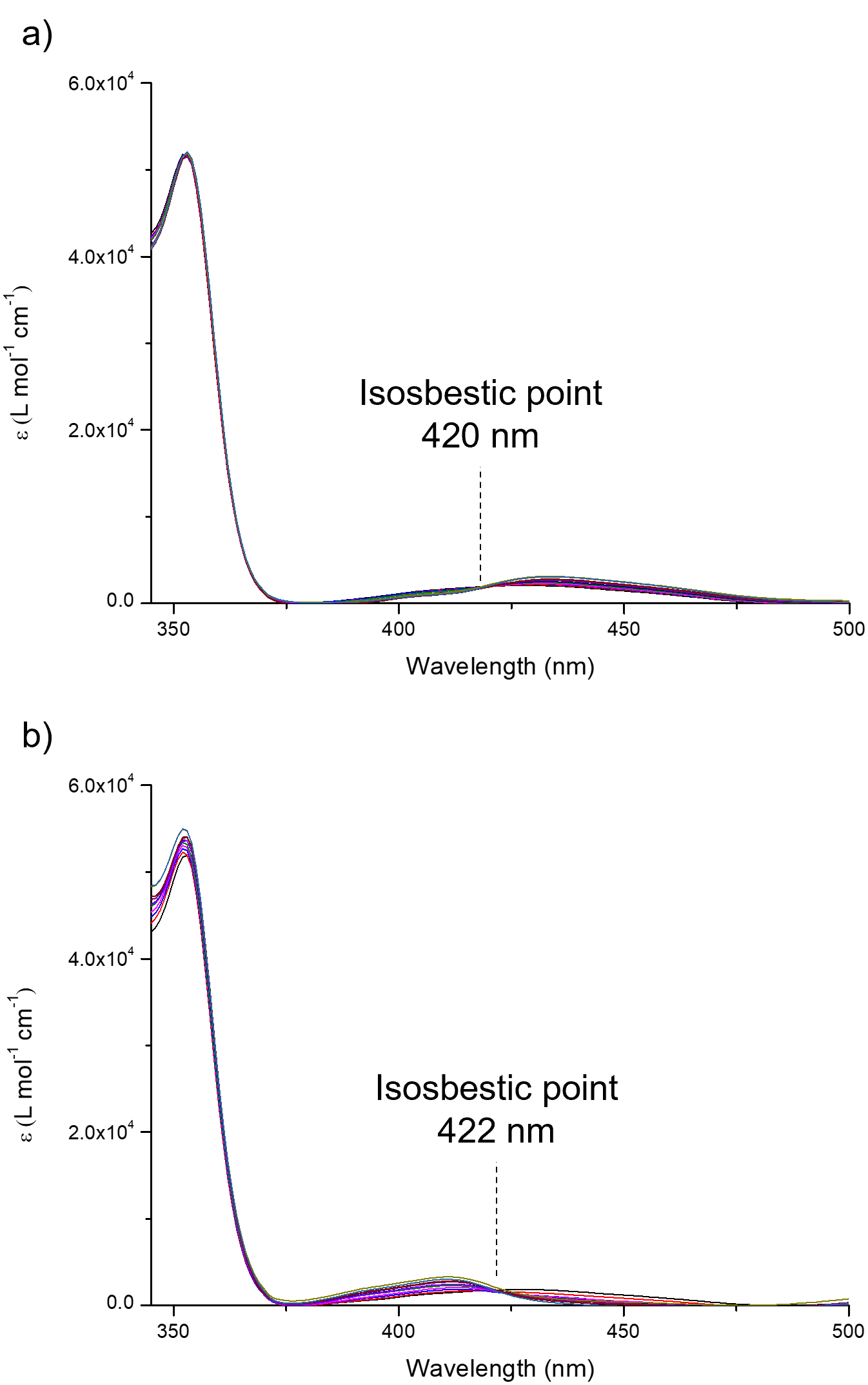


**Fig. S7.** UV-Vis absorption spectra upon addition of a) methanol and b) water to a solution of 1.0 x 10^-5^ M **FDMFA** in THF (adding 2 μL each up to 20 μL).

Comment: When methanol and water were each added to the THF solution of FDMFA up to 20 μL, as shown in Fig. S7, the maximum absorption wavelength due to the n-π* transition shifted to a longer wavelength (434 nm) for methanol and to a shorter wavelength (411 nm) for water. It seems that these two additive solvents affect the ground state energy level of FDMFA differently due to the difference in hydrogen bond acidity.
